# Annual Ambient Black Carbon Associated with Shorter Telomeres in Elderly Men: Veterans Affairs Normative Aging Study

**DOI:** 10.1289/ehp.0901831

**Published:** 2010-08-17

**Authors:** John McCracken, Andrea Baccarelli, Mirjam Hoxha, Laura Dioni, Steve Melly, Brent Coull, Helen Suh, Pantel Vokonas, Joel Schwartz

**Affiliations:** 1 Department of Environmental Health, Harvard School of Public Health, Boston, Massachusetts, USA; 2 Center of Molecular and Genetic Epidemiology, IRCCS (Istituto Di Ricovero e Cura a Carattere Scientifico) Maggiore Hospital, Milan, Italy; 3 Department of Environmental and Occupational Health, University of Milan, Milan, Italy; 4 Department of Biostatistics, Harvard School of Public Health, Boston, Massachusetts, USA; 5 Veterans Affairs Outpatient Clinic, Boston, Massachusetts, USA; 6 Department of Medicine, Boston University School of Medicine, Boston, Massachusetts, USA

**Keywords:** air pollution, biological aging, cardiovascular physiology, environmental exposure, epigenetic process, particles, traffic, vehicle emissions

## Abstract

**Background:**

Telomere length reflects biological age and is inversely associated with risk of cardiovascular disease (CVD). Ambient air pollution is associated with CVD, but its effect on telomere length is unknown.

**Objective:**

We investigated whether ambient black carbon (BC), a marker for traffic-related particles, is associated with telomere length in the Normative Aging Study (NAS).

**Methods:**

Among 165 never-smoking men from the NAS, leukocyte telomere length (LTL) was measured repeatedly approximately every 3 years from 1999 through 2006 using quantitative real-time polymerase chain reaction (qRT-PCR). BC concentration at their residences during the year before each LTL measurement was estimated based on a spatiotemporal model calibrated with BC measurements from 82 locations within the study area.

**Results:**

The median [interquartile range (IQR)] annual moving-average BC concentration was 0.32 (0.20–0.45) μg/m^3^. LTL, expressed as population-standardized ratio of telomere repeat to single-copy gene copy numbers, had a geometric mean (geometric SD) of 1.25 (1.42). We used linear mixed-effects models including random subject intercepts and adjusted for several potential confounders. We used inverse probability of response weighting to adjust for potential selection bias due to loss to follow-up. An IQR increase in annual BC (0.25 μg/m^3^) was associated with a 7.6% decrease (95% confidence interval, −12.8 to −2.1) in LTL. We found evidence of effect modification, with a stronger association among subjects ≥ 75 years of age compared with younger participants (*p* = 0.050) and statin medications appearing protective of the effects of BC on LTL (*p* = 0.050).

**Conclusions:**

Telomere attrition, linked to biological aging, may be associated with long-term exposures to airborne particles, particularly those rich in BC, which are primarily related to automobile traffic.

Telomeres are regions of noncoding DNA at the end of chromosomes that protect against structural degradation, inappropriate recombination, and end-to-end fusion of chromosomes (Blackburn 2001; Wong and Collins 2003). Telomere length declines with each successive cell division and thus serves as a measure of biological aging (Aviv 2002). Shorter telomeres are associated with greater risk of various chronic diseases, including diabetes (Fitzpatrick et al. 2007), hypertension (Fitzpatrick et al. 2007; Perez-Rivero et al. 2006; Yang et al. 2009), atherosclerosis (Benetos et al. 2004), coronary artery disease (Brouilette et al. 2007; Mukherjee et al. 2009), and heart failure (van der Harst et al. 2007).

Evidence from *in vitro* studies (Honda et al. 2001; Saretzki and Von Zglinicki 2002; von Zglinicki 2002) and human studies (Fitzpatrick et al. 2007; Saretzki and Von Zglinicki 2002) suggests that oxidative stress and inflammation accelerate telomere shortening. These findings have motivated investigation of whether environmental factors that influence oxidative and inflammatory responses also decrease telomere length. Telomeres are shorter among people who smoke (McGrath et al. 2007; Morla et al. 2006; Valdes et al. 2005), consume more processed red meat (Nettleton et al. 2008), are obese or gain weight during adulthood (Kim et al. 2009), or live a sedentary lifestyle (Cherkas et al. 2008), leading to the general conclusion that one mechanism by which these factors influence cardiovascular disease (CVD) risk is by accelerating the biological aging of cells, revealed in part by shorter telomeres.

Associations between ambient air pollution—both short-term episodes and long-term exposures—and CVD have been observed in numerous epidemiological studies, and systemic inflammation and oxidative stress are central components of hypothesized biological mechanisms (Brook et al. 2004). The mechanisms leading to the short-term effects of CVD have been investigated intensively in recent years and include increased systemic inflammation (Schwartz 2001), blood coagulation (Baccarelli et al. 2007, 2008b; Seaton et al. 1999), arterial vasoconstriction (Brook et al. 2002), alteration of autonomic control (Pope et al. 1999), and modification of epigenetic processes (Baccarelli et al. 2009). Investigation of the potential biological mechanisms underlying chronic health effects has been limited. Telomere attrition, a predictor of CVD that reflects biological aging and the cumulative effects of oxidative stress, is a plausible mediator of the chronic health effects of air pollution. One previous study found that traffic officers had shorter leukocyte telomeres than did office workers and that occupational exposures to benzene and toluene were inversely associated with telomere length (Hoxha et al. 2009). We are unaware of any study on the association between typical exposures to fine ambient particles and telomere length in a general population.

In this study, we use longitudinal data from the Normative Aging Study (NAS) to investigate whether ambient concentration of black carbon (BC), primarily representing mobile source particles, outside an individual’s home during the previous year was associated with telomere length.

## Materials and Methods

### NAS subjects

The NAS is an investigation of aging established in 1963 by the U.S. Department of Veterans Affairs, and all subjects were free of chronic disease at the time of recruitment. The present study is restricted to a subset of the 880 persons still participating between 1 January 1999 (when DNA collection began) and 31 December 2006, during which period health and risk factors evaluations were performed approximately every 3 years. The eligibility criteria for this study were never smoking and residing in Massachusetts within 40 km of a BC monitor. We focused on never-smokers because tobacco smoke is a particle-rich mixture that has many attributes and effects (e.g., inflammation) in common with ambient particles but involves a much greater dose. Because this dose is measured imperfectly by questionnaires, we felt the measurement error could be large compared with the particle doses we were examining and could obscure any association with our exposure. We identified 179 participants who met the eligibility criteria, and we obtained at least one telomere length measure on 165 (92%). The median age of these men was 74 years, and the range was 56–94 years; 43 (26%) of these subjects had coronary heart disease.

### Spatiotemporal model for mobile source particles

Concentration of BC, a surrogate for particles from gasoline- and especially diesel-powered motor vehicles, outside each subject’s home during the year before telomere measures was estimated based on a previously validated spatiotemporal model (Gryparis et al. 2007). Briefly, Gryparis et al. 2007) modeled 24-hr measures of BC based on > 6,000 observations from > 2,000 unique exposure days during the years 1999–2004 at 82 locations. Predictions were based on meteorologic and other characteristics (e.g., weekday/weekend) of a particular day, as well as measures of land use (e.g., cumulative traffic density within 100 m and percent urbanization) at each monitor location. The model also allowed for space–time interactions by including interaction terms between the temporal meteorologic predictors and the source-based geographic variables. The most important interactions of this type were mixing height (the height above the ground below which the atmosphere is well mixed) with percent urbanization and daily average wind speed with cumulative traffic density. Gryparis et al. (2007) used thin-plate splines to model residual spatial variability. They fit separate models for the warm and cold season. The prediction equation also included central monitor concentrations as a predictor to reflect average concentration for a given day. The highest predicted daily concentration level was more than three times that of the lowest predicted level, and the adjusted *R*^2^ for this model was 0.83. Monthly monitoring campaigns were conducted subsequently at 37 locations and used as a validation sample. The out-of-sample predicted *R*^2^ based on this validation sample was 0.53 for 24-hr measurements. All addresses of participants in the NAS were geocoded, and annual average BC concentration corresponding to each telomere length measurement was defined as the average BC concentration over the 365 days before the visit at which a blood sample was collected to assess the outcome.

### Telomere length measurements

We collected 7 mL of whole blood by venous phlebotomy in EDTA tubes. DNA was extracted from stored frozen buffy coat using the QiAmp DNA blood kits (Qiagen, Germantown, MD, USA) and used for leukocyte telomere length (LTL) measurement by means of quantitative real-time polymerase chain reaction (qRT-PCR) (Cawthon 2002). Relative LTL was measured by determining the ratio of the telomere (T) repeat copy number to the single-copy gene (S) copy number (T:S ratio) in a given sample and reported as relative units expressing the ratio between LTL in the test DNA and LTL in a DNA pool used to generate a standard curve in each PCR run. We used pooled DNA from 475 participants randomly selected from the NAS (50 ng for each sample) to create for each run a fresh standard curve, which ranged from 20 to 0.25 ng/μL. An eight-point standard curve, derived from serially diluted DNA pool, ranging from 30 to 0.234 ng/μL was included in each PCR plate, so that relative quantities of T and S could be determined. For each study sample, we prepared a 25-μL mixture of DNA sample (2 ng/μL) containing *Escherichia coli* DNA (15 ng/μL) used as a DNA carrier to increase PCR reproducibility; we heated these mixtures to 96°C × 10 min and then cooled them to room temperature. PCR primer sets for T and human beta-globin, taken as the reference S, as well as the PCR mix composition were previously described (Hou et al. 2009). Using a MICROLAB STARlet Robot (Hamilton Life Science Robotics, Bonaduz, Switzerland), we transferred 2 μL (2 ng/μL) DNA in 5 μL reaction mix in 384-well plates. We performed the PCR runs on a 7900HT Fast Real-Time PCR System (Applied Biosystems, Foster City, CA, USA). After PCR amplification, the specificity of the product was confirmed by dissociation curve analysis. We ran all samples in triplicates, and the average of the three T measurements was divided by the average of the three S measurements to calculate the average T:S ratio (Farzaneh-Far et al. 2008). To test the reproducibility of this method, we amplified T and S in 15 samples replicated three times on each of 3 consecutive days. The coefficient of variation for the average T:S ratio of samples analyzed over 3 consecutive days was 8.7%, similar to the reproducibility originally reported for this method (Cawthon 2002).

### Statistical analysis

Natural log-transformed LTL was the dependent variable in linear mixed effects regression models run using Proc Mixed from SAS (version 9.1; SAS Institute Inc., Cary NC, USA). To estimate the effect of annual moving-average BC concentration on LTL, we included random intercepts for each subject, thereby accounting for correlation among repeated measures of LTL, and used the empirical estimator of variance for confidence intervals (CIs) and *p*-values (Zeger and Liang 1986). In the baseline model, we included as potential confounders age at first telomere measure, the difference between this first age and the age at each measure, and year of telomere measurement. In the next model, to adjust for potential confounding, we included education level (< high school, high school graduate, < 4 years of college, 4 years of college, > 4 years of college) because we assumed it is likely to be a common cause of BC exposure and LTL. We also included body mass index (BMI), white blood cell count, percent neutrophils, percent lymphocytes, treatment with a statin medication (yes/no; then-current use based on self-report), diagnosis of diabetes (yes/no), and fasting blood glucose because we assumed these variables may share an unmeasured common cause with BC exposure and are likely to be determinants of LTL. Next, we built a model in which we also adjusted for living in an urban area, census-tract median income, and percentage of population below the poverty line (U.S. Census Bureau poverty thresholds from year 1999) as area-level covariates (U.S. Census Bureau 1999). Residential areas were defined as urban (yes/no) according to the National Atlas of the United States (U.S. Geological Survey 2009). For each continuous covariate, we evaluated the potential to further reduce confounding by including quadratic terms. We multiplied the regression coefficient for BC by the pollutant’s interquartile range (IQR) and calculated the percent difference in LTL associated with an IQR increase in BC.

The telomere measures constitute an imbalanced longitudinal data set, with subjects having varying numbers of repeated measures. Loss to follow-up may lead to selection bias if data are not missing at random, conditional on the terms included in our regression models (Rubin 1976). We repeated the above analyses after weighting follow-up observations by the inverse probability of attaining a follow-up response (Robins et al. 1995). A subject with a repeated measurement that occurred < 5 years before the end of the study (31 December 2006) was defined as complete, and a subject for whom ≥ 5 years had passed between their last observation and the end of the study was defined as lost to follow-up. In a logistic regression model, the log odds of the response indicator (response = 1, missing = 0) was predicted by covariates from the previous measurement occasion, including telomere length, annual BC, age, BMI, statin use, and education level. We then weighted the observed follow-up measures by the inverse of the estimated probabilities of obtaining a follow-up response, which gives more weight to observations that are more likely to be missing, and we assigned the earlier observations for each subject a weight of 1.

Although the advantage of using a spatiotemporal exposure model is that we can estimate BC at a subject’s residence without placing a monitor there, we acknowledge that there is uncertainty about the extent to which the model can be extrapolated across space. For the main analyses, we aimed to balance this concern against that of maintaining an adequate sample size and limited the distance to 40 km. As a sensitivity analysis, we examined the association between BC and LTL among subjects living < 10 km from one of the monitors.

Telomere shortening is related to biological aging (Aviv et al. 2009). Thus, we evaluated whether chronological age modifies the effect of annual BC on LTL by including a BC × age interaction term in the model. Because accelerated telomere shortening is associated with inflammation, we also tested for an interaction between BC and treatment with statin medications (then-current use based on self-report), which have been shown to reduce inflammation (Liu et al. 2009).

This study protocol was approved by the institutional review boards of all participating institutions. All participants gave written informed consent.

## Results

Although limited to never-smokers and almost entirely white (97%), this subgroup of men from the NAS was fairly heterogeneous according to BMI, education level, and census-tract median income (Table 1).

We obtained two LTL measurements for 90 of the 165 participants (55%) and a third measurement for 19 participants (12%); 17 (10%) of the participants died before a repeat measure could be collected, and the rest were lost to follow-up for various reasons, including refusal and relocation out of the study area. Over the 274 measures, LTL ranged from a T:S ratio of 0.31 to 2.84 and had a median 1.24 and a geometric mean (geometric SD) 1.25 (1.42). Although there was only a small decrease of 2% in geometric mean LTL between the first and second measures, the LTL was 19% shorter at the third measurement occasion compared with the second. Rather than being individuals with typically short telomeres that happened to have three measures, these 19 subjects actually had a geometric mean LTL of 1.39 at the start (vs. 1.27 for the population as a whole) that decreased to 1.02 by the third measure. Although LTL declined on average 2.5% (95% CI, −4.6% to −0.5%) with each year of within-subject change in age, we observed wide variation in the rate of change within subjects, and LTL values of some individuals increased from one measurement to the next.

**Figure 1:**
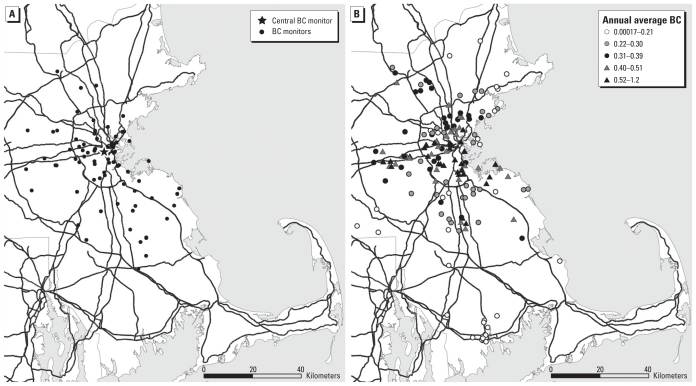
Maps of study area in eastern Massachusetts showing (*A*) locations of BC monitors used in spatiotemporal statistical model and (*B*) approximate locations of residences of NAS participants, with coding (white, gray, black) to indicate quintiles of annual moving averages of predicted daily BC concentrations, 1999–2006.

Figure 1 shows the location of the BC monitors contributing to the exposure model and residential locations along with corresponding quintile of estimated annual moving-average BC level corresponding to each subject’s first measurement occasion. BC levels tend to be higher near downtown Boston. Overall, the mean (± SD) annual BC concentration was 0.32 ± 0.20 μg/m^3^, and the mean decreased slightly with each repeated measure (mean first measure, 0.33; mean second measure, 0.30; and mean third measure, 0.26).

Adjusting for age and year, we found that an IQR (0.25 μg/m^3^) increase in annual moving-average BC concentration was associated with a 5.8% decrease (95% CI, −10.8% to −0.4%) in LTL (Table 2). After adjusting for additional potential confounders, including educational level, BMI, and several other relevant physiologic measures and indicators of health status, the association was similar, equivalent to a 5.4% decrease in LTL (95% CI, −10.0% to −0.7%) per IQR increase in BC. After also adjusting for living in an urban area and census-tract-level socioeconomic variables, the association was stronger, a 7.9% decrease in LTL (95% CI, −12.8% to −2.7%) per IQR increase in annual BC. Use of inverse probability of response weighting to reduce potential selection bias that stems from loss to follow-up resulted in a similar estimate, a 7.6% decrease in LTL (95% CI, −12.8% to −2.1%) per IQR increase in annual BC. We made these same adjustments for the sensitivity analysis and tests for interaction presented below.

Restricting the study population to the subjects living within 10 km of the nearest BC monitor (132 subjects, 215 measures) resulted in a stronger association, and an IQR increase in BC was associated with a 9.0% decrease (95% CI, −16.5% to −0.9%) in LTL.

**Figure 2:**
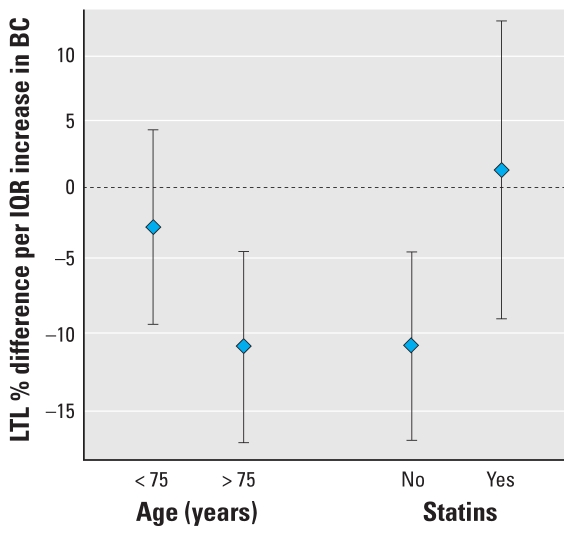
Modification of the effect of annual moving-average BC concentration on LTL by age and statin treatment: NAS, 1999–2006. Estimated percent differences (95% CIs) associated with an interquartile increase (0.25 μg/m^3^) in annual BC are shown, adjusted for baseline age; change in age between measures; year; BMI (quadratic); white blood cell count; percent neutrophils; percent lymphocytes; treatment with statin medication (yes, no); diagnosis of diabetes (yes, no); fasting blood glucose; education level (< high school, high school graduate, < 4 years of college, 4 years of college, > 4 years of college); National Atlas of the United States urban indicator for residence location, census-tract–level median income, and percentage of population below poverty line; and potential selection bias due to loss to follow-up by inverse probability of follow-up response weighting.

We found a significant interaction between annual BC and age ≥ 75 years in their effects on LTL (*p* = 0.050), and the direction of this interaction suggests that older participants were more susceptible to the effects of BC than those < 75 years of age. We also found a statistically significant interaction between BC and statin treatment (*p* = 0.050), with no evidence of an association between BC and LTL among men on statin medications. Figure 2 plots the point estimates and 95% CIs for the association between LTL and BC according to age group and statin treatment.

## Discussion

Investigating the chronic effects of ambient air pollution, we hypothesized that people exposed to higher levels of combustion particles from mobile sources would have shorter telomeres on the ends of their chromosomes. Among 165 never-smoking adults from the NAS cohort, we investigated the association between near-residence annual moving-average concentrations of BC and LTL. Analyzing a total of 270 repeated observations, we found that an IQR increase in BC was associated with a 7.6% decrease in telomere length (*p* = 0.008), adjusting for potential confounders, such as age, education level, BMI, year, several relevant aspects of medical history, living in an urban area, and census-tract-level socioeconomic variables, as well as for loss to follow-up using inverse probability of response weighting.

The biology of telomere dynamics suggests two main mechanisms by which traffic particles could influence LTL. The first is by influencing the rate of cell replication. The ends of chromosomes progressively shorten as cells undergo division because of the inability of DNA polymerase to replicate the lagging DNA strand to its terminus (Allsopp et al. 1992). Chronic inflammation, for example, is associated with increased numbers of leukocytes as well as their more rapid expenditure, both requiring a higher rate of replication of cells up the hierarchy of the hematopoietic system. Circulating leukocytes are derived from hematopoietic stem cells, so the lengths of telomeres in an individual’s leukocytes reflect the length of the telomeres in their hematopoietic stem cells, which are shortened by their lifetime of replenishing leukocytes. A second type of mechanism leading to shorter telomeres influences the extent of telomere loss during each replication. For instance, the GGG triplets on telomeres are highly sensitive to hydroxyl radicals, and oxidative stress is a major determinant of telomere shortening independent of shortening due to incomplete end replication when stem cells divide to produce new leukocytes (von Zglinicki 2002). A causal interpretation of the association between traffic particles and LTL in our study, therefore, relies on the plausibility that traffic particles could be involved in at least one of these two types of mechanisms.

Indeed, inflammation and oxidative stress are central in the current mechanistic understanding of the cardiovascular health effects of particulate air pollution (Brook et al. 2004; Chahine et al. 2007). Epidemiologic studies, human exposure experiments, animal models, and *in vitro* studies have provided evidence of inflammatory effects from short-term exposures to particles (Donaldson and Tran 2002; O’Neill et al. 2007; Rückerl et al. 2007). Inflammation may also play a role in the chronic effects of air pollution, as suggested by an analysis of the Third National Health and Nutrition Examination Survey by Chen and Schwartz (2008) showing that exposure to annual average PM_10_ (PM with aerodynamic diameter ≤ 10 μm) was associated with higher white blood cell count. Toxicologic experiments demonstrate that combustion particles promote atherosclerosis (Mills et al. 2007), widely recognized as an inflammatory disease (Ross 1999). Moreover, Künzli et al. (2005) found that a 10-μg/m^3^ increase in annual near-residence PM_2.5_ (PM with aerodynamic diameter ≤ 2.5 μm) in Los Angeles, California, was associated with approximately a 4% increase in carotid intima-media thickness, a measure of subclinical atherosclerosis. There is also clear evidence that diesel exhaust exposure causes oxidative DNA damage, and human panel studies have shown that guanine oxidation, in particular, is associated with particle exposure (Gonzalez-Flecha 2004; Risom et al. 2005). Oxidative stress mediated by PM may arise from direct contact with reactive oxygen species on the surface of particles that deposit in the lungs, soluble compounds such as transition metals or organic compounds that enter the bloodstream, or activation of inflammatory cells capable of generating reactive oxygen and nitrogen species (Risom et al. 2005).

Traffic-particle exposure, therefore, may increase both the rate of replication of hematopoietic stem cells, due to inflammation, as well as the extent of telomere loss per replication, due to oxidative stress. In our study, approximately an 8% shorter LTL was associated with an IQR increase in annual BC. Telomere shortening, which leads to endothelial dysfunction, one of the earliest features of atherosclerosis, may be an underlying cause of atherosclerosis (van der Harst et al. 2007). An important research question, therefore, becomes whether telomere shortening mediates the effects of particle-induced chronic, low-grade inflammation, and oxidative stress on the development and progression of atherosclerotic coronary artery disease. The mechanisms described above, however, fail to explain the wide temporal variability in telomere length that led to increases in LTL among some subjects rather than a monotonic decrease in length with aging, and the causes and health implications of this aspect of telomere dynamics, also observed in previous studies (Aviv et al. 2009; Ehrlenbach et al. 2009; Martin-Ruiz et al. 2005), are unclear. The small coefficient of variation (8.7%) we observed among the triplicate measures of the T:S ratios taken over 3 consecutive days suggests that these increases in LTL with age are not attributable to measurement error alone.

A key concept that has come to the fore in air pollution epidemiology is interindividual variability in susceptibility. Demographic and socioeconomic characteristics, medical history, and genetic variants have been identified as predictors of susceptibility (Baccarelli et al. 2008a; Chahine et al. 2007; O’Neill et al. 2007). In our study of annual average BC and LTL levels, we found statistically significant interactions with both age and statin treatment, with larger effect estimates among subjects ≥ 75 years of age compared with younger participants, and among those not taking statins compared with statin users. Similarly, previous studies have reported stronger associations between daily particle exposure and both inflammatory markers and heart rate variability among subjects not taking statins (O’Neill et al. 2007; Schwartz et al. 2005), as well as between annual PM_2.5_ and carotid intima-media thickness among older subjects (Künzli et al. 2005).

The spatiotemporal statistical model we used to estimate near-residence concentrations of BC was built upon measurements at 82 locations in the greater Boston, Massachusetts, area. We took the pollutant measurements that informed the model during varying time intervals from January 1999 through September 2004. The model eliminates a substantial amount of measurement error that would have been incurred if estimates had been derived based on exposure levels collected at a central site monitor, due to the large amount of spatial heterogeneity in BC levels. This is evident when one compares the increase in out-of-sample *R*^2^ for the model-based estimates (0.53) with that from a model based on central site measurements alone (0.10). However, because we have used relatively simple structures to approximate complex spatiotemporal interactions, there is still some error associated with these estimates, which will likely attenuate our estimated health effects toward the null (Gryparis et al. 2009). However, the fact that we are aggregating predicted daily BC levels over yearly periods to estimate chronic effects serves to lessen the effect of this form of model misspecification, because much of the measurement error is due to random fluctuations that average out over longer periods. For instance, Gryparis et al. (2009) considered the impact of using 9-month integrated predictions from this same model to assess the association between birth weight and BC exposure during gestation. Using newly developed measurement error correction techniques, these authors estimated that the attenuation resulting from using the predicted 9-month values is on the order of 20%. Indeed, when we restricted our study population to those living within 10 km of a BC monitor, our effect estimates were slightly larger, which may be attributable to a reduction in exposure measurement error due to spatial extrapolation.

BC is used in our study as a surrogate for traffic pollution and may not itself be causally related to LTL, so the estimated effect of traffic pollution may be biased if BC is not a reliable surrogate for the overall toxicity of this pollutant mixture. Although the use of BC as a surrogate for traffic-related particles is supported by the predictive value of cumulative traffic density in the spatiotemporal model we used for near-residence ambient BC concentrations (Gryparis et al. 2007), as well as other studies that have shown ambient BC to be associated with proximity to automobile traffic (Kim et al. 2004; Patel et al. 2009), there are other sources of ambient BC, such as wood burning (LaRosa et al. 2002), so BC is an imperfect surrogate for particles from automobiles. The direction of the bias introduced by this type of measurement error is unknown because the relative toxicity of the other sources is unknown, but we assume that the bias would be small because automobile traffic is the major source of ambient BC in the study area (Gryparis et al. 2007).

Another limitation of our study is that we used relative telomere length as a surrogate for absolute telomere length. Although these two measures have been shown to be moderately correlated, prediction of absolute lengths from the relative measures involves a nonzero intercept term (Ehrlenbach et al. 2009), so the percent changes we report are specific to relative LTL measures and are not associated with equivalent proportional changes in absolute LTL. This nonzero intercept in the relationship between these two types of measures, rather than differences in measurement error between the PCR and Southern blot methods, also explains the much wider coefficient of variation of relative LTL compared with that of absolute LTL.

As in most longitudinal studies, there was imbalance in the number of repeated measures per subject, here largely due to loss to follow-up. Although the similar results after using inverse probability of response weighting suggest that a spurious association due to loss to follow-up was unlikely, this approach relies on correctly specifying a model for the dropout process, so it does not rule out potential selection bias due to dependent censoring. It is also possible that we did not include or correctly adjust for confounders of the relationship between BC and LTL. A particularly important concern when evaluating effects of air pollution across a metropolitan area is that higher levels of air pollution are associated with lower socioeconomic status (O’Neill et al. 2007), a determinant of many factors likely associated with shorter telomeres. Although we adjusted for education level, census-tract median income, and percentage of population below the poverty line, residual and unmeasured confounding by socioeconomic status is possible. Additionally, we did not measure factors such as psychological stress, lack of physical exercise, and environmental tobacco smoke (ETS), all suspected to be associated with shorter telomere length and potential confounders if they are associated with exposure to traffic particles in the NAS cohort (Cherkas et al. 2008). Schikowski et al. (2008) found that distance to major roads and ETS exposure were both associated with lower socioeconomic status in the Ruhr area of Germany, which suggests that confounding by ETS is an important concern for ambient BC studies in general. However, we assume that ETS exposure would be fairly low among the population of elderly, male never-smokers included in our analyses and that any such bias would be reduced by the adjustment for socioeconomic variables in our models. Moreover, among the overall NAS cohort under follow-up during the same study period (605 subjects), we found no association between active smoking and near-residence annual average BC concentration (0.01 μg/m^3^ higher BC among active smokers; *p* = 0.531), which suggests that ETS, presumably having a similar geographic distribution as active smoking, is unlikely to be an important confounder (data not shown).

In addition to ETS, there are other important indoor sources of BC, such as cooking and candle burning (LaRosa et al. 2002), that we did not take into account in our ambient BC predictions. If we were investigating the biological effects of total BC exposure, this would result in substantial measurement error and would be an important limitation of our study. However, we used ambient BC as a surrogate for traffic-related particles, in which case exclusion of BC of indoor origin actually reduces measurement error. Nevertheless, BC from indoor sources presents a possible source of confounding if ambient BC and indoor-generated BC are correlated. An ongoing exposure assessment study including indoor BC and sulfate-based penetration ratios shows that the correlation between BC of outdoor origin and BC of indoor origin is low in NAS homes (*r* = −0.13; data not shown). Therefore, confounding of the association between ambient BC and telomere length by BC of indoor origin is unlikely to be an important source of bias in our study.

Our findings suggest that higher annual average exposure to traffic-related air pollution is associated with shorter leukocyte telomeres among the elderly. Telomere attrition is a measure of biological aging that explains some interindividual variation in risk of atherosclerosis and coronary artery disease and may play an important role in the chronic health effects of airborne particles, particularly those rich in BC, which are primarily related to automobile traffic.

## Correction

Steve Melly was omitted from the list of authors in the manuscript originally published online. His name has been added here.

## Figures and Tables

**Table 1:** Characteristics of NAS subjects: male never-smokers from eastern Massachusetts, 1999–2006.

Variable	All visits	First visit	Second visit	Third visit
No. of measures	274	165	90	19
Age [years (mean ± SD)]	74.6 ± 7.1	73.6 ± 7.1	75.9 ± 7.0	76.5 ± 6.6
Education level [*n* (%)]				
< High school	10 (4)	6 (4)	4 (4)	0 (0)
High school	83 (30)	45 (27)	32 (36)	6 (32)
< 4 years of college	61 (22)	39 (24)	17 (19)	5 (26)
4 years of college	59 (22)	39 (24)	17 (19)	3 (16)
> 4 years of college	61 (22)	36 (22)	20 (22)	5 (26)
Census-tract median income, $1000s (mean ± SD)	62.3 ± 19.8	63.3 ± 20.4	60.5 ± 19.6	60.8 ± 13.6
Living in an urban area [*n* (%)]	164 (60)	99 (60)	55 (61)	10 (53)
BMI [kg/m^2^ (mean ± SD)]	27.6 ± 3.7	27.7 ± 3.9	27.4 ± 3.6	26.8 ± 3.0
Waist circumference [cm (mean ± SD)]	98.6 ± 9.4	98.5 ± 9.0	99.1 ± 9.9	103.0 ± 10.3
Coronary artery disease [*n* (%)]	82 (30)	43 (26)	31 (34)	8 (42)
Fasting blood glucose [mg/dL (mean ± SD)]	105 ± 24	106 ± 27	104 ± 18	104 ± 17
Diabetes [*n* (%)]	32 (12)	17 (10)	12 (13)	3 (16)
Statin treatment [*n* (%)]	106 (39)	46 (28)	48 (53)	12 (63)
Leukocyte count [cells/cm^3^ (mean ± SD)]	6.3 ± 2.0	6.1 ± 1.6	6.3 ± 2.0	7.5 ± 3.8
Neutrophils [% (mean ± SD)]^a^	62 ± 8	62 ± 7	62 ± 8	61 ± 12
Lymphocytes [% (mean ± SD)]^a^	26 ± 7	26 ± 7	26 ± 7	28 ± 13
Telomere length				
Median	1.25	1.27	1.23	1.12
Geometric mean (geometric SD)	1.25 (1.42)	1.27 (1.34)	1.25 (1.54)	1.02 (1.41)

a Neutrophil and lymphocyte percentages of total leukocytes obtained for 270 visits.

**Table 2:** Association between annual moving-average BC exposure and LTL, expressed as difference in LTL per IQR (0.25 μg/m^3^) increase in BC concentration (*n* = 165 subjects).

Model	Observations (*n*)	LTL percent change (95% CI)	*p*-Value
Adjusted for age and period^a^	274	−5.8% (−10.8% to −0.4%)	0.036
Plus individual-level covariates^b^	270	−5.4% (−10.0% to −0.7%)	0.026
Plus urbanity and census-tract socioeconomic status^c^	270	−7.9% (−12.8% to −2.7%)	0.003
Plus inverse probability weighting^d^	270	−7.6% (−12.8% to −2.1%)	0.008

a Adjusted for baseline age, change in age between measures, and year.

b Additionally adjusted for BMI (quadratic), white blood cell count, percent neutrophils, percent lymphocytes, treatment with statin medication (yes, no), diagnosis of diabetes (yes, no), fasting blood glucose, and education level (< high school, high school graduate, < 4 years of college, 4 years of college, > 4 years of college).

c Additionally adjusted for National Atlas of the United States urban indicator for residence location, census-tract-level median income, and percentage of population below poverty line.

d Additionally adjusted for potential selection bias due to loss to follow-up by assigning inverse probability of follow-up response weights to follow-up observations during the latter part of the study period (from January 2002 to December 2006). In a logistic model, the response indicator was predicted by covariates from the previous measurement occasion, including telomere length, annual BC, age, BMI, statin use, and education level.
