# Correction: Annual Ambient Black Carbon Associated with Shorter Telomeres  in Elderly Men: Veterans Affairs Normative Aging Study

**DOI:** 10.1289/ehp.0901831-erratum

**Published:** 2010-08-17

Steve Melly was omitted from the list of authors in the manuscript originally published online. His name has been added here.

